# Purinergic Signaling in Mast Cell Degranulation and Asthma

**DOI:** 10.3389/fphar.2017.00947

**Published:** 2017-12-22

**Authors:** Zhan-Guo Gao, Kenneth A. Jacobson

**Affiliations:** Molecular Recognition Section, Laboratory of Bioorganic Chemistry, National Institute of Diabetes and Digestive and Kidney Diseases, National Institutes of Health, Bethesda, MD, United States

**Keywords:** purinergic signaling, adenosine receptors, P2Y receptors, P2X receptors, mast cell degranulation, asthma, allergy, bronchoconstriction

## Abstract

Mast cells are responsible for the majority of allergic conditions. It was originally thought that almost all allergic events were mediated directly only via the high-affinity immunoglobulin E receptors. However, recent evidence showed that many other receptors, such as G protein-coupled receptors and ligand-gated ion channels, are also directly involved in mast cell degranulation, the release of inflammatory mediators such as histamine, serine proteases, leukotrienes, heparin, and serotonin. These mediators are responsible for the symptoms in allergic conditions such as allergic asthma. In recent years, it has been realized that purinergic signaling, induced via the activation of G protein-coupled adenosine receptors and P2Y nucleotide receptors, as well as by ATP-gated P2X receptors, plays a significant role in mast cell degranulation. Both adenosine and ATP can induce degranulation and bronchoconstriction on their own and synergistically with allergens. All three classes of receptors, adenosine, P2X and P2Y are involved in tracheal mucus secretion. This review will summarize the currently available knowledge on the role of purinergic signaling in mast cell degranulation and its most relevant disease, asthma.

## Introduction

**FIGURE 1:**
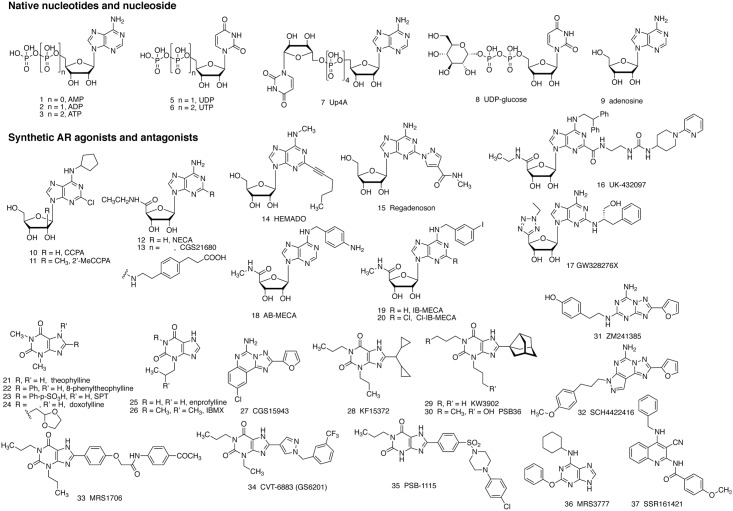
Structure of native agonists of the purinergic receptors, including both P2Rs (1–8) and ARs (9), and structures of agonist (10–20) and antagonist (21–37) ligands developed for the ARs, as described in the text. Compounds 24 – 26 inhibit PDEs, but are weaker in inhibiting ARs.

It is known that purinergic signaling is involved in various immune responses (Cekic and Linden, 2016; Cronstein and Sitkovsky, 2017). However, its role in mast cell degranulation, which leads to hypersensitivity reactions in response to environmental factors, is not fully understood. There are three subfamilies of receptors, 7 P2X receptor (P2XR) subunits (combined into functional trimeric channels), 8 P2Y receptors (P2YRs) and 4 adenosine receptors (ARs), that respond to purine nucleosides and purine (or pyrimidine) nucleotides (Jacobson and Gao, 2006; Chen et al., 2013; Burnstock and Boeynaems, 2014). Adenosine 5′-triphosphate (ATP, compound 3 in **Figure 1**) is abundant in mast cells, stored in granules and secreted upon activation. ATP acts via P2X receptors (P2XRs), which are ligand-gated cation channels, to induce mast cell degranulation (Bulanova and Bulfone-Paus, 2010). In general, ATP is considered a major damage-associated molecular pattern molecule (DAMP) in the immune system, and one of its principle mechanisms is by activating the P2X7R (Di Virgilio and Vuerich, 2015). Other nucleotides, such as adenosine 5′-diphosphate (ADP) 2, uridine 5′-diphosphate (UDP) 5, uridine 5′-triphosphate (UTP) 6, Up4A 7 and UDP-glucose (UDPG) 8, act mainly via P2Y receptors which are coupled to G proteins (Jacobson et al., 2015; Gao et al., 2010a,b, 2013). Purine nucleosides, especially adenosine 9, released under stress conditions, are demonstrated to be involved in many allergic conditions, particularly, the pathogenesis of asthma and the subsequent chronic obstructive pulmonary diseases (COPD) (Adriaensen and Timmermans, 2004; Barnes, 2011). Both adenosine and allergens can cause bronchoconstriction (Cushley et al., 1984; Rafferty et al., 1987; Fozard, 2003; Hua et al., 2013b). Adenosine 5′-monophosphate (AMP) 1 also induces bronchoconstriction in asthmatic patients, and this compound, which forms adenosine *in situ*, is used in inhalation challenge testing (Isogai et al., 2017). Additionally, adenosine, ATP, and allergens can induce mast cell degranulation independently or synergistically (Nunomura et al., 2010; Hua et al., 2013a).

AR antagonists, theophylline 21 and enprofylline 25, have long been used in the clinic, particularly for asthma (Schultze-Werninghaus and Meier-Sydow, 1982). The mechanism of action of these xanthines was initially thought to be via the inhibition of phosphodiesterases (PDEs), and they are now considered to also act via the antagonism of one or several subtypes of ARs (Marquardt et al., 1978; Pauwels and Joos, 1995; Fozard, 2003; Barnes, 2011). It should be noted that adenosine-induced bronchodilation is possibly mediated via the A_2B_AR, whereas bronchoconstriction occurs via the A_1_AR. Antagonism of the A_1_AR causes bronchodilation, while blockade of the A_2B_AR causes bronchoconstriction (which will be discussed later). The simple methylxanthines, e.g., theophylline, often antagonize both A_1_ and A_2B_ARs thus producing a mixed effect, although the overall effect is bronchodilation in most cases. It should also be kept in mind that inhibition of PDE3 and PDE4 should produce a net effect similar to that of activation of the A_2B_AR, i.e., elevation of 3′,5′-cyclic adenosine monophosphate (cAMP) levels in smooth muscle cells. However, upon examination of the dose response curves for action of simple xanthines, the antagonism of ARs begins to occur at lower concentrations than PDE inhibition (Daly and Fredholm, 1998). An increase in cAMP leads to activation of protein kinase A (PKA) and exchange protein directly activated by cAMP (EPAC), which phosphorylate target proteins, leading to the modulation of myosin activity and eventually relaxation of smooth muscle. The A_2B_AR is also known to induce intracellular Ca^2+^ mobilization in many types of cells including smooth muscle cells leading to the relaxation of tracheal smooth muscle, which is often independent of Gs-protein and cAMP. It is important to understand the physiological roles and the signaling mechanisms involved in order to develop purinergic agonists and antagonists with appropriate selectivity and efficacy. Several P2YRs, e.g., P2Y_13_ and P2Y_14_, (Gao et al., 2010a,b, 2013) and P2XRs, e.g., P2X4 and P2X7 (Yoshida et al., 2017), are also recently demonstrated to be mediators and/or potentiators of mast cell degranulation.

**FIGURE 2:**
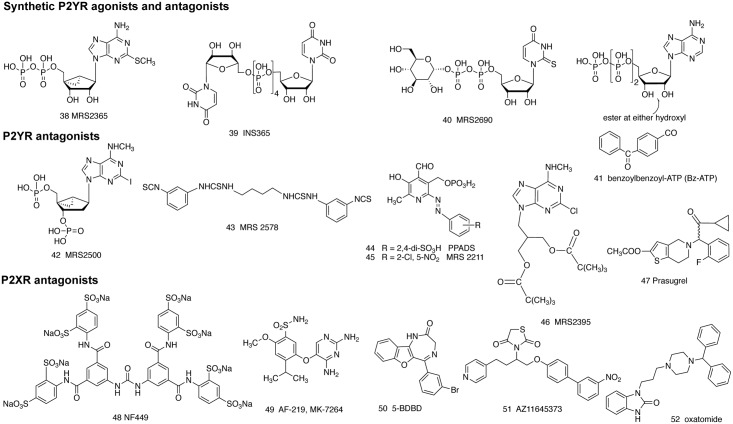
Structure of various synthetic agonist (38–41) and antagonist (42–52) ligands for the P2YRs and P2XRs, as described in the text. Compound 52 was introduced as an antihistamine and later found to block the P2X7R.

This review will first summarize the currently available knowledge related to the role of adenosine, P2Y and P2X receptors in mast cell degranulation. We will then analyze the therapeutic rationale and potential mechanisms of AR, P2Y, and P2X receptor ligands in asthma, particularly in bronchoconstriction and tracheal mucus secretion. Methylxanthines, e.g., theophylline 21, enprofylline 25, and doxofylline 24 are used in asthmatics for the alleviation of bronchoconstriction and trachea mucus secretion (Barnes, 2011). Two inhaled, selective AR ligands with anti-inflammatory actions, i.e., A_2A_AR agonist UK432097 16 (for COPD) and mixed A_2A_AR agonist/A_3_AR antagonist GW328267X 17 (for asthma and allergic rhinitis) failed to show efficacy in clinical trials, but there were complicating pharmacokinetic factors (Mantell et al., 2010). Selective A_2B_AR antagonist CVT-6883 34 was under development for asthma (Zablocki et al., 2005). P2Y_2_R agonists uridine 5′-triphosphate 6 (UTP) and INS365 (compound 39 in **Figure 2**) have been in clinical trials for patients with cough due to its potential in airway mucus clearance (Noone et al., 1999; Kellerman, 2002). Orally active P2X3R antagonist MK-7264 49 (gefapixant, AF-219) is under clinical investigation for the treatment of idiopathic chronic cough, asthma, pulmonary fibrosis and other conditions (clinicaltrials.gov) [accessed October 15, 2017]. Thus, all three sub-families of receptors activated by purine nucleosides or nucleotides, are potential targets for asthma and some other allergic conditions.

## Review Contents: Roles of Four AR Subtypes in Mast Cell Degranulation, Mucus Secretion and Bronchoconstriction

It has been known for decades that inhaled adenosine induces bronchoconstriction in asthmatics and COPD patients, but not in non-asthmatics (Cushley et al., 1984). Exercise-induced asthma is often accompanied by increases in plasma adenosine (Fozard, 2003). Both adenosine deaminase (ADA) and AR antagonist theophylline 21 can block adenosine-induced bronchial hyperresponsiveness (Rothstein, 1980). However, it is still not fully understood which AR subtype is actually involved in the antiasthmatic effects of methylxanthines (Barnes, 2011). It is important to establish the precise roles of adenosine and AR subtypes in mast cell degranulation, bronchoconstriction and mucus secretion, and develop appropriate AR subtype-selective agonists/antagonists for asthma and COPD.

## *In Vitro* Studies of Degranulation Using Mast Cell Lines

### RBL-2H3 Cells

RBL-2H3 rat basophilic cells are a useful model for studies of degranulation. Ali et al. (1990) have shown that a non-selective adenosine agonist, NECA 12, acts synergistically with antigen in RBL-2H3 mast-like cells via a novel AR in a pertussis toxin (PTX)-sensitive manner. This novel AR was later cloned and defined as A_3_AR (Zhou et al., 1992). Collado-Escobar et al. (1990) reported that the widely used glucocorticoid dexamethasone down-regulates IgE-receptor-mediated signals but up-regulates A_3_AR-mediated signals in RBL-2H3 cells, suggesting A_3_AR involvement in inflammation and mast cell function. Ramkumar et al. (1995) showed later that dexamethasone increases the expression of both A_3_AR and G proteins in RBL-2H3 cells which contributes to the enhanced response to adenosine. Jin et al. (1997) reported that, in addition to adenosine, inosine, which was known to bind to the rat A_3_AR (Jacobson et al., 2017), also stimulates degranulation in RBL-2H3 cells. Thus, results from these earlier studies suggest that adenosine and its analogs, acting via the A_3_AR, can stimulate degranulation on their own, enhance the effect of antigen to stimulate degranulation via FcεRI receptor, and may offset the anti-inflammatory effects of glucocorticoids, such as dexamethasone, suggesting the anti-allergic potential of the A_3_AR antagonists.

However, unlike the results from studies using RBL-2H3 cells, Auchampach et al. (1997) showed that in canine mast cells which express A_1_AR, A_2B_AR, and A_3_AR, degranulation is mediated by the A_2B_AR, rather than the A_3_ or A_1_ARs. NECA-stimulated degranulation is not PTX-sensitive and is blocked by enprofylline 25, a slightly A_2B_AR selective antagonist (*K*_i_ = 7 or 4.7 or 19.8 μM at human A_2B_AR), with weaker effects on human A_1_AR (42 μM), A_2A_AR (32 μM), and A_3_AR (65 μM) (Müller and Jacobson, 2011). Auchampach et al. (1997) suggest that A_1_AR and A_3_AR might involve a mast cell function other than degranulation in this specific cell type. However, there was no further report since then on the role of the A_2B_AR in canine mast cell degranulation.

### HMC-1 and LAD2 Human Mast Cell Lines

Two human mast cell lines, HMC-1 (Butterfield et al., 1988) and LAD2 (Kirshenbaum et al., 2003), have been used for the study of mast cell function. HMC-1 cell line is often not considered as a good model for studying mast cell degranulation due to the low expression level of high-affinity IgE receptor (Guhl et al., 2010), but it has some other mast cell functions. Feoktistov and Biaggioni (1995) demonstrated that HMC-1 cells express both A_2A_ and A_2B_ARs. NECA 12, but not A_2A_AR-selective agonist CGS21680 13, induced interleukin (IL)-8 production in HMC-1 mast cells in an enprofylline-sensitive manner, suggesting a possible role of the A_2B_AR in mast cell function. In the simulated tumor microenvironment, contact with cancer cells induces HMC-1 cells to upregulate IL8 secretion, and this effect is dependent on released adenosine activating the A_3_AR (Gorzalczany et al., 2017).

The LAD2 cell line can be used as a model for the study of mast cell degranulation. LAD2 cells highly express FcεRIα and FcεRIγ, and antigens can induce a robust release of histamine (Guhl et al., 2010). It is suggested that connective tissue-type and mucosal-type mast cells are developed via distinct pathways, and tryptase/chymase expression can be considered as an indication of the maturity of mast cells (Ma et al., 2008; Guhl et al., 2010). Guhl et al. (2010) reported that tryptase and chymase expression is low in LAD2 cells in comparison to that in the primary skin mast cells, although much higher than in HMC-1 cells. Nevertheless, Leung et al. (2014) were able to examine the role of ARs in degranulation of human LAD2 mast cells, which express A_2A_, A_2B_, and A_3_ but not A_1_ARs. The non-selective agonist NECA alone induced a small but significant stimulation of β-hexosaminidase (β-hex) release. Further, NECA increased both antigen and C3a-stimulated degranulation. The authors suggested that more than one AR subtype is involved in degranulation. Thus, there is a difference in AR expression profile and roles of ARs in various types of mast cells. Receptor expression level may play a critical role in mast cell activation and release of both newly synthesized cytokines and chemokines and stored mediators that are implicated in mast cell mediated allergic and inflammatory reactions in asthma. It seems that the differences between LAD2 and HMC-1 in terms of degranulation are mainly related to the expression level of FCεRI, tryptase and chymase, and to a much lesser extent related to histamine content or c-Kit expression (Guhl et al., 2010). The role of adenosine to induce or enhance degranulation may also be related to the AR expression in various types of mast cells. The use of primary mast cells is needed for the characterization of the roles of various ARs.

## *In Vitro* Studies of Degranulation Using Primary Mast Cells

### Murine Primary Mast Cells

The role of adenosine receptors in mast cells degranulation was first reported in primary rat mast cells (Marquardt et al., 1978). Both adenosine and inosine were found to potentiate degranulation (Marquardt et al., 1978). Theophylline, at concentrations of 1–100 μM, blocks the potentiating effect of adenosine without affecting other mast cell functions (Marquardt et al., 1978), suggesting that the beneficial effects of theophylline in bronchial asthma is possibly via an AR subtype, but it is not clear if the A_3_AR is involved, as methylxanthines are weak at the rat or mouse A_3_AR (Jacobson and Gao, 2006). Möller et al. (2003) reported that activation of bone marrow derived mouse mast cells (BMMC) with NECA caused the release of β-hex, although to a lesser extent than antigen-induced release via FcεRI. The specific AR subtype involved in degranulation was not reported in that study, although A_1_AR expression and survival was found enhanced upon FcεRI activation. Nunomura et al. (2010) suggested a mechanism of synergistic degranulation response in BMMC is via FcεRI and ARs. The FcεRI beta-chain (FcRbeta) was found to be a critical element in a synergistic mast cell degranulation response through FcεRI and ARs. Furthermore, phosphoinositide 3-kinase (PI3K)-signaling through FcRbeta immunoreceptor tyrosine-based activation motifs (ITAM) is a crucial participant in augmentation of FcεRI-mediated degranulation by adenosine, although the specific AR subtype involved in degranulation was not investigated. Leung et al. (2014) also found that NECA enhanced antigen-induced degranulation in BMMC. Zhong et al. (2003) established primary murine lung mast cell cultures and demonstrated the expression of A_2A_, A_2B_, and A_3_ ARs on murine lung mast cells. The authors suggest that the A_3_AR plays an important role in adenosine-mediated murine lung mast cell degranulation. Thus, adenosine or its analogs are clearly demonstrated to induce and/or enhance degranulation in primary murine mast cells, although it remains to be established if one AR or multiple AR subtypes are involved.

### Human Primary Mast Cells

Gomez et al. (2011) reported FcεRI-induced degranulation is different in primary human lung and skin mast cells after exposure to adenosine. Human lung mast cells were found to express the A_3_AR threefold higher than human skin mast cells. Low concentrations of adenosine or an A_3_AR agonist was found to potentiate FcεRI-induced degranulation of human lung mast cells but not that of skin mast cells, in a PTX-dependent way. The authors suggest that A_3_AR, as a potentiator of FcεRI-induced degranulation, may involve a bronchoconstrictive response to adenosine in asthmatics, but not dermatologic allergy responses. The results also suggested that the AR expression level is related to the extent of AR-mediated degranulation. Yip et al. (2011) reported the dual and opposing modulation by A_1_ and A_2B_ARs of anti-IgE induced histamine release from primary human mast cells derived from progenitor stem cells. By using multiple selective AR agonists (CCPA 10, A_1_; 2′-MeCCPA 11, A_1_; HEMADO 14, A_2A_; Cl-IB-MECA 20, A_3_) and antagonists (PSB-36 30, A_1_; SCH442416 32, A_2A_; MRS1706 33, A_2B_; PSB-1115 35, A_2B_; MRS3777 36, A_3_), the authors showed that inhibition of human mast cell activation is a mechanism for A_1_AR antagonists, but not A_2B_AR antagonists. Hua et al. (2011) found that IL-4, a cytokine that is involved in airway inflammation, increased expression of the A_2B_AR in human umbilical cord blood-derived mast cells. The authors suggest that Th2 cytokines in the asthmatic lung may upregulate AR expression on airway mast cells to promote increased responsiveness to adenosine, which may explain at least in part why asthmatic lungs are more sensitive to adenosine than normal lungs or skin. Thus, results from primary human mast cells indicate that multiple AR subtypes are possibly involved in degranulation. The A_3_AR and possibly A_1_AR induce and/or potentiate, whereas the A_2B_AR may inhibit degranulation in human mast cells. Thus, results from primary human mast cells are not completely consistent with those from cell lines, such as LAD2, HMC-1, or canine mast cells, especially concerning the role of the A_2B_AR. It is also noted that from the aspect of degranulation, the A_1_AR and A_2A_AR most likely are not major players, although isolated reports suggest in certain cell types A_1_AR plays a contributory role. The A_2A_AR, a largely anti-inflammatory AR, may be involved in many mast cell functions other than degranulation.

## *In Vivo* Studies of Degranulation

In addition to the studies from mast cell lines and primary mast cells described above, by studying vasoconstriction of hamster cheek pouch arterioles, Shepherd et al. (1996) showed that both adenosine and its metabolite, inosine, can cause vasoconstriction *in vivo* by stimulation of mast cell degranulation via the hamster A_3_AR. Reeves et al. (1997) reported that the A_3_AR promotes degranulation of rat mast cells both *in vitro* and *in vivo*. Fozard et al. (1996) studied A_3_AR activation in anesthetized Sprague-Dawley rats (using SPT 23 at a dose that blocks rat A_1_, A_2A_ and A_2B_ ARs, but not A_3_). The authors suggest that the A_3_AR activation results in rapid mast cell degranulation, which plays a key role in A_3_AR-mediated hypotension in rat. Thus, the role of the A_3_AR in mast cell degranulation *in vivo* in rodents is consistent with findings from RBL-2H3 cells and primary murine mast cells. However, concerning the effects of dexamethasone, Hannon et al. (2002) reported that adenosine-induced mast cell degranulation in rat *in vivo* is suppressed by dexamethasone, which is in contrast to the findings by using RBL-2H3 cells (Collado-Escobar et al., 1990), suggesting potentially both pro- and anti-inflammatory roles of the A_3_AR.

In an effort to study the role of the A_3_AR in mast cell degranulation and development of A_3_AR antagonists for allergic conditions especially for asthma (to evaluate the pharmacological effects of human A_3_AR antagonists in mice or rats), Yamano et al. (2005) generated A_3_AR-humanized mice, in which the mouse A_3_AR gene was replaced by its human homolog, the authors found that human A_3_AR can activate intracellular Ca^2+^ mobilization but not the mouse PI3K-γ signaling pathway. Antigen-dependent degranulation was not potentiated by the A_3_AR agonist in the mast cells from A_3_AR-humanized mice, suggesting the complexity of the A_3_AR signaling and function in mast cells and in different species. The use of A_3_AR agonists 19 and 20 in clinical trials has not revealed any serious adverse effects (Jacobson et al., 2017).

The role of the A_3_AR in mast cell degranulation and inflammation has been explored using A_3_AR knockout (KO) mice. Salvatore et al. (2000) demonstrated that adenosine and the A_3_AR agonist, Cl-IB-MECA 20, potentiate antigen-dependent degranulation of BMMCs from wild-type (WT) but not A_3_AR(-/-) mice, as measured by β-hex release. The authors also showed that A_3_AR plays a role in both pro- and anti-inflammatory responses. Tilley et al. (2003) identified A_3_AR- and mast cell-dependent and -independent components of adenosine-mediated airway responsiveness in mice. The authors indicate that mouse airway responses to aerosolized adenosine are largely dependent on A_3_AR activation with a significant contribution from mast cells, and that activation of additional ARs on other cell types may also contribute to adenosine-induced airway responsiveness *in vivo*. Tilley et al. (2000) showed that both adenosine and inosine increase cutaneous vasopermeability by activating A_3_AR on mast cells. Using mice deficient in the A_3_AR, the authors showed that increases in cutaneous vascular permeability induced by adenosine or its metabolite inosine are mediated through the A_3_AR. Also, adenosine does not increase vascular permeability in mast cell-deficient mice. This response is independent of activation of FcεRI, by antigen, as adenosine is also increases permeability in FcεRI beta-chain-deficient mice. Highly specific A_3_AR agonists caused hypothermia in mice via peripheral mast cell degranulation, although the body temperature reduction was dependent on a central histamine H_1_ receptor (Carlin et al., 2016). This study made use of AR KO mice (A_1_AR, A_3_AR and combined A_1_AR/A_3_AR), a non-brain-penetrant A_3_AR agonist and mast cell depletion. Thus, *in vivo* studies suggest a role of the A_3_AR in degranulation, independent of antigen activation of the high-affinity IgE receptor. In addition to using A_3_AR KO mice, Zhong et al. (2003) showed that lung mast cells in ADA-deficient mice degranulated robustly with the elevated adenosine present. ADA prevented the accumulation of lung adenosine as well as mast cell degranulation, suggesting that this process was dependent on elevated lung adenosine levels. Consistent with this, treatment of ADA-deficient mice with non-selective AR antagonists attenuated degranulation by 30–40%. These studies are consistent with the ability of adenosine generated *in vivo* to activate ARs and thereby enhance lung mast cell degranulation. Thus, the role of the A_3_AR in mast cell degranulation has been well established. However, there has not been report about the role of the A_3_AR in asthmatic patients, although *in vitro* studies in human mast cells suggested a role. This could be partly due to the fact that other events beyond degranulation, such as bronchoconstriction and trachea mucus secretion need more immediate attention for patients with asthma and are possibly more related to the A_1_ and A_2B_AR mechanisms, which will be discussed later in this manuscript.

Concerning the role of AR subtypes other than the A_3_AR in mast cell degranulation, the A_2B_AR is the most studied. In a variety of studies including in primary human or mouse mast cells and in receptor KO mice, the A_2B_AR has been demonstrated to inhibit rather than mediate mast cell degranulation. Yip et al. (2011) reported that activation of the human mast cell A_2B_AR inhibits anti-IgE induced release of histamine, while A_1_AR agonists potentiated mast cell activation. Hua et al. (2007) reported that mice deficient in the A_2B_AR showed enhanced mast cell activation. Basal levels of cAMP were reduced in BMMCs from A_2B_AR KO mice and the influx of extracellular calcium through store-operated calcium channels following antigen activation was increased. A_2B_AR KO mice also are more sensitive to IgE-mediated anaphylaxis. The authors suggest that the A_2B_AR can act in concert to attenuate mast cell responsiveness following antigen exposure. Thus, A_2B_AR agonists rather than antagonists can be considered as a therapy for asthma. Hua et al. (2013a) reported that the two Gs-coupled A_2A_ and A_2B_ARs differentially limit antigen-induced mast cell activation. By comparing mast cell responses of mice with various combinations of AR KOs, they showed that AR agonists can modulate mast cell degranulation and induction of cytokine production both *in vitro* and *in vivo*. A_2B_AR was identified as the principal subtype attenuating mast cell degranulation; however, both A_2A_ and A_2B_AR need to be activated to inhibit cytokine synthesis.

Zaynagetdinov et al. (2010) reported that, unlike the role of the A_2B_AR in acute inflammation, genetic deletion of A_2B_AR reduced allergen-induced chronic pulmonary inflammation, accompanied by fewer bronchoalveolar lavage eosinophils and lower peribronchial eosinophilic infiltration. Allergen-induced IL-4 release in airways was observed in WT, but not in A_2B_AR KO mice. Ryzhov et al. (2008b) demonstrated that BMMCs in A_2B_AR KO mice display two distinct phenotypes. One effect is enhanced antigen-induced degranulation, consistent with an inhibitory role of A_2B_AR in degranulation as reported by Hua et al. (2013a). The other effect observed in A_2B_AR KO mice is loss of NECA-induced increases of IL-13 leading to vascular endothelial growth factor (VEGF) secretion. However, Ryzhov et al. (2008a), by using A_2B_AR KO mice, demonstrated that A_2B_AR upregulates the proinflammatory cytokine IL-6. Thus, it seems A_2B_AR activation can induce secretion of several proinflammatory cytokines, which apparently contradicts an anti-inflammatory role for the A_2B_AR. Indeed, it has been proposed that an A_2B_AR antagonist rather than agonist would be suitable for potential use in asthma, based on the findings that A_2B_AR induced IL-8 secretion by an enprofylline-sensitive mechanism in HMC-1 cells (Feoktistov and Biaggioni, 1995), a mast cell line that does not degranulate but has some other mast cell functions. The A_2B_AR is involved in degranulation of canine BR mastocytoma cells which can be blocked by enprofylline (Auchampach et al., 1997). However, this conclusion needs to be examined more carefully, considering the fact that A_2B_AR activation inhibits degranulation in primary human and murine mast cells. Inhibition of PDEs (Barnes, 2011) and activation of histone deacetylase (HDAC) (Barnes, 2011) are often described as a major mechanism for methylxanthines in the treatment of asthma. The inhibition of the A_2B_AR probably produces side effects rather than a desired therapeutic effect.

The role of the A_2A_AR in mast cell degranulation was explored in a number of earlier studies. Hughes (1984) showed that adenosine and NECA can either inhibit or potentiate IgE-dependent histamine release by human lung mast cells in suspension, depending on the time sequence. However, the A_2B_AR and A_3_AR had not yet been cloned or defined at that time, thus it is not clear which specific AR is involved in inhibition or enhancement. Lohse et al. (1987) showed that adenosine and its analogs enhance the release of histamine from rat peritoneal mast cells. The authors suggest that an A_2_ AR is involved in adenosine-induced enhancement of histamine release, but it is not clear if it is through the A_2A_AR or A_2B_AR, as the AR subtypes were not yet defined. Marquardt et al. (1994) showed that A_2A_AR is not involved in BMMC degranulation, as an A_2A_AR-specific agonist failed to enhance mast cell mediator release. Gomez et al. (2013) showed that adenosine specifically inhibited FcεRI but not through the A_2A_AR. Rork et al. (2008) reported that A_2A_AR activation in the isolated, perfused mouse heart inhibits degranulation of resident cardiac mast cells to limit the extent of infarction. The authors found that CGS21680 significantly reduced mast cell degranulation in WT but not in A_2A_AR KO mice. Suzuki et al. (1998) suggested that adenosine acts via the A_2A_AR to inhibit FcεRI-mediated release of tryptase from primary human mast cells, as this inhibitory effect can be mimicked by CGS21680 and blocked by A_2A_AR/A_2B_AR antagonist ZM241385 31.

In summary, of the 4 ARs in mast cell degranulation, it seems that A_1_AR plays a minor role, and A_2A_AR, although overall anti-inflammatory, either does not have an effect or plays an inhibitory role in mast cell degranulation. However, A_2B_AR has prominent proinflammatory and anti-inflammatory roles depending what is measured. A_2B_AR may induce proinflammatory cytokines from some types of cells but inhibit mast cell degranulation both in human and murine mast cells, both *in vitro* and *in vivo*, although isolated studies of mast cell showed that it may also cause degranulation (e.g., in canine mast cells, Auchampach et al., 1997). The A_3_AR has also been demonstrated to be both pro- and anti-inflammatory. However, in terms of its role in mast cell degranulation, most pieces of evidence suggest that A_3_AR mediates mast cell degranulation, but in a species-dependent fashion (Carlin et al., 2016). Thus, A_2B_AR and A_3_AR, which inhibits and stimulates, respectively, are the two major AR subtypes involved in mast cell degranulation. Additionally, ARs are also involved in the function of other granulocytes such as neutrophils, basophils, and eosinophils, which are also related to release of inflammatory mediators albeit to a lesser extent compared with mast cells (Barletta et al., 2012), but this is not the main focus of the current review.

Asthma and COPD are probably the most relevant conditions related to adenosine release and subsequent AR activation that are primarily initiated by mast cell degranulation, which is followed by bronchoconstriction and mucus secretion. We have mainly examined the role of ARs in mast cell degranulation in the above sections. We will then summarize and analyze the roles of 4 ARs in the mucus secretion and bronchoconstriction in the following sections.

## Role of ARS in Mucus Secretion

Although the mechanisms of action are still debatable, methylxanthines, such as theophylline and enprofylline, have been used for asthma treatment for almost a century presumably due to their effect on mast cell degranulation, bronchoconstriction and airway mucus clearance. Mucus hypersecretion is an important contributor to airway obstruction. The action of methylxanthines on mucus clearance may complement their effects on mast cell degranulation and bronchoconstriction in asthmatic patients (Wanner, 1985; Ziment, 1987; Wang et al., 2016); the mechanism of methylxanthines have been proposed to be via PDEs, HDAC, and ARs (Barnes, 2011). In an earlier study, Wagner et al. (1996) reported effects of several xanthines as PDE inhibitors, i.e., theophylline 21, enprofylline 25, and 3-isobutyl-1-methylxanthine 26 (IBMX), on tracheal mucus secretion in rat and found that they stimulate mucus secretion with EC_50_ values of 690, 400, and 46 μM, respectively. This may suggest a possible mechanism as mixed PDE inhibition and AR antagonism, or interpreted as the antagonism of multiple ARs, as methylxanthines are non-selective AR antagonists. As will be discussed in the following sections, blockade of the A_1_AR and A_2B_AR may have a respective positive and negative impact on trachea mucus clearance. Increasing attentions have been paid on the roles of ARs in mucus clearance in recent years including the use of KO animals, although the role of individual AR subtypes is still controversial. In the following section, we briefly summarize the roles of ARs in mucus clearance.

McNamara et al. (2004) showed that mucin 2 (MUC2) expression increased in response to adenosine in cultured airway epithelial cells. The authors suggest that adenosine in combination with inflammatory cytokines stimulates asthmatic airway mucin production. The results were consistent with suggested use of antagonists of A_1_AR, calcium-activated chloride channel regulator 1 (CLCA1), and epidermal growth factor receptor (EGFR) in asthma treatment. A_1_AR antagonists contribute to airway mucus clearance. Mohsenin et al. (2007) showed that genetic ablation of the A_2A_AR in ADA-deficient mice enhanced pulmonary inflammation, mucin production, and angiogenesis. Thus, A_2A_AR agonists should contribute to airway clearance. Rollins et al. (2008) demonstrated that activation of the A_2B_AR contributes to mucus clearance. Hua et al. (2013b) showed that adenosine increased mucus clearance via both A_2A_ and A_2B_ ARs. In both A_3_AR KO mice (Young et al., 2006) and ADA-deficient mice (Young et al., 2004), A_3_AR activation increases airway mucin secretion in response to allergen challenge. In summary, A_2A_ and A_2B_AR agonists or A_1_ and A_3_AR antagonists may contribute to trachea mucus clearance.

## Roles of ARS in Bronchoconstriction

As methylxanthines can inhibit PDEs, activate HDAC (i.e., theophylline), and activate ryanodine receptors, as well as antagonize ARs, many of their therapeutic effects in asthma, especially their use against bronchoconstriction, have often been ascribed to non-adenosine mechanisms, such as the inhibition of PDEs (Barnes, 2011). Theophylline’s therapeutic effect has been suggested to be due to the activation of HDAC (Donnelly and Rogers, 2003).

Inhaled adenosine induces bronchoconstriction in asthmatic patients but not in healthy subjects (Cushley et al., 1984; Rorke and Holgate, 2002). Theophylline was found more potent in blocking adenosine-induced than histamine-induced bronchoconstriction suggesting most likely an AR- but not PDE-mediated mechanism. It seems that adenosine-induced bronchoconstriction of isolated sensitized lung tissues is via the release of three mediators, i.e., histamine, cyclooxygenase products and leukotrienes (Martin and Broadley, 2002), as none of the mediators alone is responsible for the constriction. Fozard (2010) summarized apparent contradictions about the role the A_3_AR in bronchoconstriction, using sensitized Brown Norway rats. Alfieri et al. (2012) found that A_1_AR expression on smooth muscle cells is increased on bronchi of sensitized Wistar rats challenged with allergen, suggesting that the A_1_AR is responsible for bronchial hyperresponsiveness to adenosine. Hua et al. (2007) demonstrated that adenosine-induced bronchoconstriction in mice is mediated via the A_1_AR. Ponnoth et al. (2010) demonstrated using allergic WT and A_1_AR KO mice that this receptor is systemically proinflammatory and increases airway hyperresponsiveness. Use of DNA antisense against the A_1_AR in a rabbit model of asthma suggested that receptor subtype may promote bronchoconstriction (Nyce and Metzger, 1997). Two A_1_AR antagonists, KF15372 28 and KW3902 29 significantly inhibited the NECA-induced bronchoconstriction in an *in vivo* rat model. Pauwels and Joos (1995) showed that the A_1_AR is possibly involved in adenosine-induced bronchoconstriction based on the order of bronchoconstrictor potency of adenosine analogs. Mikus et al. (2013) examined the effects of a novel A_3_AR antagonist, SSR161421 37 on bronchoconstriction. In ovalbumin presensitized guinea pigs, SSR161421 (IV or PO) inhibited antigen-induced contractions in isolated tracheal muscles that were enhanced by agonist AB-MECA 18 and also reduced bronchoconstriction *in vivo*. In addition to blocking AR agonist-induced enhancement, SSR161421 significantly decreased antigen-induced contraction. However, this compound has not been extensively evaluated in other models. Thus, antagonists of the A_1_AR and possibly the A_3_AR may be beneficial for treatment of bronchoconstriction.

Pauwels and Joos (1995) demonstrated the lack of bronchoconstriction activity of CGS21680 13, and suggested that the A_2A_AR is not involved in adenosine-induced bronchoconstriction. The A_2A_AR-selective antagonist KF17837 (structure not shown) had no activity on adenosine-induced bronchoconstriction. Regadenoson 15 has been demonstrated to be safe to use in patients with mild to moderate COPD and asthma, although it is recommended that Regadenoson should be avoided in patients with severe bronchial asthma at this time (Golzar and Doukky, 2014). A_2A_AR agonist Binodenoson (structure not shown) was also shown to be well tolerated in humans without significant bronchoconstriction or pulmonary consequences (Murray et al., 2009). Thus, A_2A_AR does not seem to play a major role in adenosine-induced bronchoconstriction. Breschi et al. (2007) characterized the role of ARs in the contractility modulation of guinea-pig airway smooth muscle in normal and pathological settings. The authors found that the non-selective agonist NECA 12, relaxed tracheal muscles in preparations from normal and sensitized animals that were pre-exposed to histamine to induce contraction, and this effect was completely blocked by an A_2B_AR antagonist, MRS1706 33. Administration of NECA or adenosine to normal animals inhibited histamine-mediated bronchoconstriction. Adenosine plasma levels were demonstrated significantly higher in sensitized than normal animals. The authors suggest that the A_2B_AR is responsible for the relaxing effects of adenosine on guinea-pig airways. A_2B_AR, but not A_2A_AR activation contributes to the relaxing of adenosine-induced bronchoconstriction. Thus, development of selective A_2B_AR agonists are a potential future direction for the treatment of asthma.

In summary, all four AR subtypes are to some extent involved in three aspects related to asthma, mast cell degranulation, trachea mucus secretion, and bronchoconstriction. To develop drugs for asthma and COPD, it is important to consider ligands with appropriate agonist or antagonist activity and subtype selectivity at certain AR subtypes, e.g., compounds with A_2B_AR agonist and A_1_AR antagonist activity. Although not yet available, allosteric A_2B_AR agonist modulators in theory could be potentially be a novel attractive therapy for asthma, due to the site- or event- specific nature of allosteric modulators. Also, as adenosine is a ubiquitous bronchoconstrictor, the prevention of adenosine accumulation in airways by modulators of ADA, AK and adenosine transporters should also be considered as future anti-asthmatic therapy. The importance of ARs in asthma was also highlighted by a recent report that the inhalation of a novel glucocorticoid receptor agonist GW870086X (structure not shown) protects against adenosine-induced bronchoconstriction in asthma (Leaker et al., 2015). Both methylxanthines and glucocorticoids have been suggested to act via HDAC, one of the converging points for inflammation control. Also, although not reviewed in this manuscript, both adenosine and allergen may regulate immune response via other types including T cells (Erdmann et al., 2005; Mukherjee and Zhang, 2011). In human asthma, initial exposure to allergen or adenosine may induce T cell-dependent stimulation or inhibition of the immune response mediating the production of IgE and cytokines. Subsequent allergen exposure may then activate mast cells to release histamine and leukotrienes.

## P2YRs

### P2YRs in Mast Cell Degranulation

Several P2Y receptor subtypes have recently been demonstrated to be mediators of mast cell degranulation. Jaffar and Pearce (1990) showed that ATP-induced release of prostaglandin D2 and histamine from rat serosal mast cells was inhibited by antagonists of both P2X and P2Y receptors. Schulman et al. (1999) suggested that ATP-enhanced histamine release from human lung mast cells are possibly via the P2Y_1_ and P2Y_2_ receptors. However, Lee et al. (2001) suggested that ATP-induced histamine release in rat peritoneal mast cells is via a P2X receptor rather than a P2Y subtype. UDPG 8, a glycosyl donor in the biosynthesis of carbohydrates, acting at the P2Y_14_R was first identified as a mediator of degranulation in RBL-2H3 mast cells as indicated by β-hex release (Gao et al., 2010b), suggesting a potential novel therapeutic target for allergic conditions. The role of P2Y_14_R was further confirmed using human LAD2 mast cells (Gao et al., 2013). All eight P2YRs were expressed at variable levels in LAD2 cells. Gene expression levels of ADP receptors, P2Y_1_, P2Y_12_, and P2Y_13_Rs, are similar, but it seems only P2Y_13_ plays a major role in degranulation. Although P2Y_11_ and P2Y_4_Rs are highly expressed (three–fivefold of P2Y_1_R), they do not seem to have a role in degranulation. Both UDPG 8 and MRS2690 40, enhanced C3a-induced β-hex release, which was inhibited by a P2Y_14_ antagonist, specific P2Y_14_R siRNA and PTX, suggesting a role of P2Y_14_R activation in promoting human mast cell degranulation. The involvement of P2Y_1_ and P2Y_6_Rs in degranulation is negligible. The enhancement by ADP and ATP appears mediated via multiple receptors. In a separate study using RBL-2H3 cells it was demonstrated that, both P2Y_1_ and P2Y_13_Rs are highly expressed. Native agonist ADP was two orders of magnitude less potent than the P2Y_1_-selective agonist MRS2365 38 in inducing intracellular Ca^2+^ mobilization; however, ADP reached the same maximal efficacy as MRS2365. ADP-induced β-hex release was PTX-sensitive and antagonized by a selective antagonist of the P2Y_13_R, i.e., MRS2211 45, but not by MRS2500 42. This pharmacological profile suggested a mechanism dependent on Gi-coupled P2Y_13_R but not a Gq-coupled P2Y_1_R. ADP-mediated intracellular calcium mobilization and β-hex release were found to be via P2Y_1_ and P2Y_13_Rs, respectively, indicating selective P2Y_13_R antagonists might be useful as therapeutic agents for various allergic conditions. (Gao et al., 2010a). Gendaszewska-Darmach et al. (2016) recently showed that nucleoside 5′-O-monophosphorothioates are weak antagonists of the P2Y_14_R and blocked antigen-induced RBL-2H3 mast cell degranulation enhanced by UDPG. Hundreds of genetic variants are thought to contribute to asthma risk by modulating gene expression. Ferreira et al. (2017), using gene-based analysis, identifies four putative novel asthma risk genes, two of which are P2Y receptors, P2Y_13_R and P2Y_14_R, highlighted the importance of these two receptors. In a recent study, although not the focus of the present review and not in mast cells, Nakano et al. (2017) showed that uridine 5′-diphosphate (UDP) promoted IgE-dependent degranulation, blocked by antagonist MRS2578 43, suggesting inhibition of P2Y_6_R may also be a potential anti-asthma therapy.

### P2YRs in Bronchoconstriction

Flores-Soto et al. (2011) suggested that ATP induces tracheal muscle contraction indirectly via epithelial P2Y receptors and prostaglandins release. Basoglu et al. (2017) found that adenosine, AMP and ATP all induced bronchoconstriction in asthmatic patients, but via different mechanisms, i.e., their respective ARs and P2Y and/or P2X receptors. Lussana et al. (2015) examined the role of the P2Y_12_R in asthmatic patients, and suggested that the P2Y_12_R antagonist prodrug prasugrel 40 may reduce the bronchial inflammatory burden, and thus may contribute to asthma treatment. Leukotriene antagonists montelukast and pranlukast have been demonstrated to antagonize the P2Y_6_R (Mamedova et al., 2005). Brüser et al. (2017) recently reported that prostaglandin (PGE)_2_-glycerol ester acts as an agonist at the P2Y_6_R, further suggesting a potential role of the P2Y_6_R in asthma.

### P2YRs in Mucus Secretion

In asthma and COPD, airway mucus hypersecretion typically leads to mucostasis and plugging of the airways by mucus. ATP release in the airways is known to be elevated in COPD, and has been demonstrated to exacerbate inflammation by activating P2Y or P2X receptors. Sabater et al. (1999) showed that inhaled P2Y_2_R agonists can increase lung mucus clearance in sheep. The purinoceptor P2Y_2_R agonist diquafosol (INS365 39) has been in clinical trials to increase mucus clearance (Kellerman, 2002; Donnelly and Rogers, 2003). Button et al. (2013) reported that changes in mechanical strain is regulated by ATP and adenosine acting via P2YRs or ARs proportional to mucus hydration in airway epithelia. Shishikura et al. (2016) showed that the extracellular ATP increases MUC5AC expression and release, mainly as an autocrine agonist of the P2Y_2_R. Shirasaki et al. (2015) used MRS2395 46, an uncharged P2Y_12_R antagonist, to partially inhibit the LTE4-induced release of MUC5AC protein in the airway. The authors suggest that role of LTE4 in allergic mucus secretion partially might involve activation of P2Y_12_R. P2Y_1_R immunoreactivity was found within the respiratory epithelium and submucosal glandular tissue. P2Y_2_R immunoreactivity was localized to the mucus-secreting cells within the vomeronasal organ (VNO) (Gayle and Burnstock, 2005). Lau et al. (2011) showed that three leukotriene antagonists, i.e., montelukast, pranlukast, and zafirlukast, inhibit P2Y_6_R agonist UDP-induced ion transport in human bronchial epithelia. Thus, several P2YR subtypes play a role in mucus secretion.

In summary, multiple subtypes of P2YRs are potentially involved in degranulation, bronchoconstriction and mucus secretion. It is important to develop appropriate P2YR-selective ligands targeting all three functions related to asthma.

## P2XRs

### P2XRs in Degranulation

Rossi et al. (1992) reported interactions between high-affinity IgE receptors and ATP receptors on immature murine mast cells. Both antigen and ATP had significant effects on intracellular calcium in cells. Wareham et al. (2009) demonstrated that three subtypes of ATP receptors, P2X1, P2X4, and P2X7Rs, were identified in both the LAD2 human mast cell line and in primary human lung mast cells. Yoshida et al. (2017) studied the role of ATP in degranulation using BMMC cells, and found that both P2X4 and P2X7Rs are involved in the regulation of BMMC degranulation. P2X7R but not P2Y4 activation induced degranulation on its own. Activation of the P2X4R significantly potentiated the degranulation induced by antigen, although it does not induce degranulation on its own. Interestingly, ATP synergistically enhanced A_3_AR mediated degranulation. Thus, ATP and adenosine may induce or enhance degranulation via multiple targets synergistically. It is suggested that P2X7R antagonists are potentially attractive anti-allergic agent (Yoshida et al., 2015). Interestingly, the antihistamine oxatomide 45 has been reported to act as a P2X7R antagonist (Yoshida et al., 2015), suggesting potentially dual antagonism. Wareham and Seward (2016) showed that P2X7R activation in human mast cells by either ATP or BzATP 41 induces pronounced increases in intracellular calcium and degranulation, which are inhibited by the selective P2X7R antagonist AZ11645373 51, or by removing extracellular calcium. P2X1R activation in human mast cells also induces calcium influx, which is significantly inhibited by antagonists PPADS 44 and NF449 48. P2X1R activation does not trigger degranulation by itself. The authors indicate that P2X7R, compared with P2X1R and P2X4R, may play a more significant role in contributing to the degranulation of mast cells. Additionally, a role of the P2X3R has also been demonstrated in murine mast cells (Bulanova et al., 2009).

### P2XRs in Bronchoconstriction

The role of ATP and P2X receptors in bronchoconstriction has been extensively studied (Basoglu et al., 2005; Weigand et al., 2012; Pelleg et al., 2016). ATP plays a major role in obstructive airway diseases (Pelleg et al., 2016). Studies in animal models and in COPD and asthma patients have detected increased ATP release in the lungs, which can affect multiple surrounding cell types to increase inflammation, bronchoconstriction, and cough (Pelleg et al., 2016). Most of these effects of ATP are mediated by P2X and/or P2Y receptors. It has recently been reported that P2X3R antagonists are promising for the alleviation of chronic cough (Pelleg et al., 2016). Basoglu et al. (2017) found that ATP and adenosine have opposite effects on capsaicin challenge in asthmatic patients. Asthmatic patients showed hypersensitivity to AMP and ATP, but AMP does not mimic the effects of ATP and the effects of ATP are not mediated by adenosine, suggesting adenosine and ATP act via ARs and P2X receptors, respectively. Gui et al. (2011) showed that the dinucleotide Up4A-induced tracheal contraction was blocked by a P2X antagonist diinosine pentaphosphate (structure not shown). Montaño et al. (2013) found that ATP-induced contraction of tracheas from guinea pigs was potentiated by pretreatment with histamine, but blocked by antagonists of P2X and P2YRs, and inhibitors of COX-1 and COX-2. Oguma et al. (2007) found that ATP, via the P2X receptor, increased the sensitivity of other inducers to induce contraction of airway smooth muscle. Nagaoka et al. (2009) demonstrated that the P2X4R is involved in the contraction of airway smooth muscle cells.

### P2XRs in Trachea Mucus Secretion

The mucociliary system in the body is responsible for clearing inhaled particles and pathogens from the airways, one of the most potent of which is extracellular ATP which acts by releasing calcium ions from internal stores via the P2YRs and by activating calcium influx via the P2XRs. Several P2XRs were found localized to various tissue types present within the nasal cavity. P2X3R immunoreactivity was localized in the primary olfactory neurons located both in the olfactory epithelium and VNO and also on subepithelial nerve fibers in the respiratory region. P2X5R was found in the squamous, respiratory and olfactory epithelial cells of the rat nasal mucosa. P2X7R was also expressed in epithelial cells, suggesting an association with epithelial turnover (Gayle and Burnstock, 2005). ATP signaling has been demonstrated to be critical in maintaining proper mucus hydration of airways (Button et al., 2013). Excessive sodium salt is known to exacerbate chronic coughing. Ma et al. (1999) show that, in airway ciliated cells, extracellular sodium ions specifically and competitively inhibit an ATP-gated channel that is permeable to calcium ions, and thereby attenuate ATP-induced ciliary motility. The authors suggest that mucus clearance might be improved in chronic bronchitis and asthma by decreasing the sodium concentration of the airway surface. Chen et al. (2016) investigated the effects of P2X4R in a murine experimental asthma model, and suggested that ATP-P2X4R signaling may not only contribute to airway inflammation, but it may also contribute to airway remodeling in allergic asthma. ATP was found to enhance the allergic reaction, which was attenuated by the P2X4R antagonist, 5-BDBD 50 (Chen et al., 2016).

Thus, several P2XRs are involved in mast cell degranulation, mucus secretion, and bronchoconstriction. ATP, acting at the P2X7R may induce degranulation its own, and synergize with adenosine and allergen, suggesting a critical role in asthma.

The roles of adenosine, P2X and P2Y receptors have been extensively investigated in both immune and non-immune cells (Jacobson and Gao, 2006; Chen et al., 2013; Burnstock and Boeynaems, 2014; Jacobson et al., 2015). Interactions among receptors for nucleosides, nucleotides, and other allergic mediators in immune and non-immune cells have been explored. For example, Pinheiro et al. (2013) showed that histamine induced release of ATP from human subcutaneous fibroblasts. Oguma et al. (2007) showed that ATP enhanced the methacholine-induced contractile response in airway smooth muscle. Montaño et al. (2013) found that ATP-induced tracheal contraction was potentiated by histamine and blocked by inhibitors of COX-1 and COX-2.

Although asthma is the major disease most relevant to the purinergic signaling, other allergic conditions have also been reported to be related to purinergic signaling. For example, Weber et al. (2010) demonstrated that the P2X7R is essential for extracellular ATP release in the response of skin to allergen exposure. Thus, P2X7R antagonists might be considered for the prevention of allergic contact dermatitis.

## Conclusion

Despite the many current asthma and COPD therapies, all drugs have some drawbacks. For example, long acting β-adrenergic agonists were suggested not to be used alone in patients with asthma (Billington et al., 2017). In addition, asthma in a significant proportion of patients remains uncontrolled; thus, more novel and newer drugs are needed for its treatment. All four AR subtypes, and several P2XR and P2YR subtypes are involved in mast cell degranulation, bronchoconstriction, and tracheal mucus secretion (**Table 1**). There are opportunities to develop appropriate ligands for the treatment of asthma by targeting one or several of these three classes of receptors. Adenosine and ATP both can induce degranulation by themselves and enhance antigen-induced degranulation, suggesting a critical role in asthmatics. Compounds with A_2B_AR agonist activity or A_1_AR and A_3_AR antagonist activity, and agonists of P2Y_2_R or antagonists of P2Y_13_R, P2Y_14_R, P2X3R, P2X4R, and P2X7R should be beneficial for the treatment of asthma. Also, in addition to receptors, targeting purinergic degradation cascade, such as ADA, AK and nucleotidases, could also be an attractive approach to controlling mast cell degranulation, mucus secretion, and bronchodilation. Finally, considering the mechanisms of action, it seems that selective A_2B_AR agonists, A_1_AR and/or possibly A_3_AR antagonists, methylxanthines that lack A_2A_ and A_2B_ antagonist activity, and P2X7R antagonists should be particularly useful for the treatment of asthma.

**Table 1:** Roles of ARs, P2XRs and P2YRs in mast cell degranulation, bronchoconstriction and airway mucus secretion.

Receptor	Mast cell degranulation	Bronchial contraction	Tracheal mucus secretion	Reference
A_1_	ND	+	+	McNamara et al., 2004; Ponnoth et al., 2010
A_2A_	ND (-)	ND	-	Rollins et al., 2008
A_2B_	-	-	-	Breschi et al., 2007; Hua et al., 2007, 2013b; Yip et al., 2011;
A_3_	+	+	+	Young et al., 2006; Gomez et al., 2011
P2Y_2_	ND	ND	+	Kellerman, 2002; Donnelly and Rogers, 2003
P2Y_13_	+	ND	ND	Gao et al., 2010a
P2Y_14_	+	ND	ND	Gao et al., 2010b; Gao et al., 2013
P2X_4_	+	+	+	Nagaoka et al., 2009; Chen et al., 2016; Yoshida et al., 2017
P2X_7_	+	ND	ND	Wareham and Seward, 2016; Yoshida et al., 2017


*+, induce; -, inhibit. ND, not clearly demonstrated (see complete reference list for more information). ATP can induce mast cell degranulation, mucus secretion, and bronchoconstriction, which are reported in many publications, but the roles of specific receptor subtypes involved have not been unambiguously demonstrated so far except for P2X4 and P2X7. The specific roles of P2Y_*1*_, P2Y_*2*_, P2Y_*4*_, P2Y_*6*_, P2Y_*11*_, P2Y_*12*_, P2X1, P2X2, P2X3, P2X5, and P2X6 receptors in degranulation have not yet been unambiguously demonstrated in mast cells, although many of them are involved in some other mast cell functions and functions of many other types of immune and non-immune cells.*

## Author Contributions

Both authors listed have made a substantial, direct and intellectual contribution to the work, and approved it for publication.

## Conflict of Interest Statement

The authors declare that the research was conducted in the absence of any commercial or financial relationships that could be construed as a potential conflict of interest.
